# Surgical Deconstruction of Recurrent Basilar Apex Aneurysms Refractory to Endovascular Treatment

**DOI:** 10.7759/cureus.116008

**Published:** 2026-09-09

**Authors:** Nicholas P Derrico, Madhav Sankhyan, Thomas R Hemphill, Alessio Iacoangeli, Gustavo Luzardo

**Affiliations:** 1 Neurosurgery, University of Mississippi Medical Center, Jackson, USA; 2 Neurological Surgery, Azienda Ospedaliero Universitaria delle Marche, Torrette, ITA

**Keywords:** endovascular aneurysm repair, intracranial aneurysm, microsurgical aneurysm clipping, neuro interventional, posterior circulation aneurysm

## Abstract

Treating aneurysms of the basilar apex that have recurred despite endovascular treatment presents a difficult clinical situation. Retreatment by endovascular means carries a high risk of requiring complicated endovascular constructs, incomplete results and recurrence, and worsening symptoms of mass effect, whereas surgical disconnection presents an opportunity for a safe, immediate, and durable resolution. We present two cases of microsurgical interruption of the basilar artery immediately below the apex as a treatment for these refractory aneurysms. The angiographic presence of a posterior communicating artery and the intraoperative identification of the perforator-free zone are described, highlighting this intervention as a safe and durable treatment strategy for recurrent aneurysms of the basilar apex.

## Introduction

Runaway aneurysms are those that exhibit progressive or even accelerated enlargement, recanalization, or mass effect despite adequate initial endovascular treatment. These aneurysms of the basilar tip present a serious management challenge and a considerable risk of rupture. Taking all comers, aneurysms deemed suitable for observation in the International Study of Unruptured Intracranial Aneurysms had a 6.6% cumulative risk of rupture over 5 years, whereas those deemed necessary to undergo treatment by surgical or endovascular means had rupture risks of 5.5% and 14.9%, respectively [1]. As these aneurysms enlarge, their risk of rupture dramatically increases from 0.23-0.46% per year for small aneurysms to 6.9-100% per year in studies of large and giant aneurysms [2]. First-line endovascular treatment is also often not durable. Rates of angiographic recanalization approach 24.5%, even in cohorts with 87.8% initial endovascular occlusion, almost twice the rate of recanalization for anterior circulation aneurysms [3,4]. In studies of repeated recanalization after a second treatment, posterior circulation aneurysms have a 6.1-times greater hazard of repeat recanalization than anterior circulation aneurysms [5].

Treatment strategies for basilar tip aneurysms have evolved considerably over time. The first reported clipping of a basilar tip aneurysm was by Drake CG in 1961, when he described his experience with four patients, two of whom survived their ordeal [6]. He went on to describe the largest series to date involving multiple approaches to indirect surgical deconstruction of the aneurysm when primary clipping of the dome was not possible [7]. Deconstruction of the vasculature, that is, permanent alteration of flow within a vascular territory by sacrifice of the parent vessel, has long been recognized as a valid strategy for aneurysm control. However, over the last several decades, the emergence and expansion of endovascular techniques have shifted the dominant treatment paradigm for basilar apex aneurysms toward endovascular management [8]. Often, this includes coiling with or without stent assistance, endosaccular flow disruptors, and, more recently, flow-diverting intraluminal devices or stents. Aneurysms that fail to be controlled with these treatments present a challenge to the treatment team: whether to continue endovascular strategies that have failed the patient in the past or pursue new treatment options.

Here, we present two cases of patients with aneurysms of the basilar apex that were refractory to initial endovascular treatment and were subsequently managed with surgical disconnection of the basilar artery immediately below the superior cerebellar arteries. We discuss the surgical technique and the anatomic nuances that must be recognized to make the intervention possible with minimal morbidity.

## Case presentation

Patient 1 is a 50-year-old smoker with a history of multiple intracranial aneurysms, including prior rupture of another aneurysm, and had a broad-based 8 × 8 mm basilar tip aneurysm that incorporated the left P1 segment of the posterior cerebral artery. She was initially managed with stent-assisted coiling of the aneurysm, which resulted in >90% occlusion of the aneurysm. She was managed on aspirin and clopidogrel for three months and then transitioned to aspirin alone. The patient was unreliable in attending follow-up, and at an 18-month follow-up angiogram, the aneurysm had already recanalized and grown without symptoms (Figure 1). Given the aneurysm's growth and her prior hemorrhage from another aneurysm, she was evaluated for retreatment. Of note, this patient’s proximal anatomy was unfavorable, and an attempt at endovascular coiling was made, but we were unable to access the aneurysm due to tortuosity.

**Figure 1:**
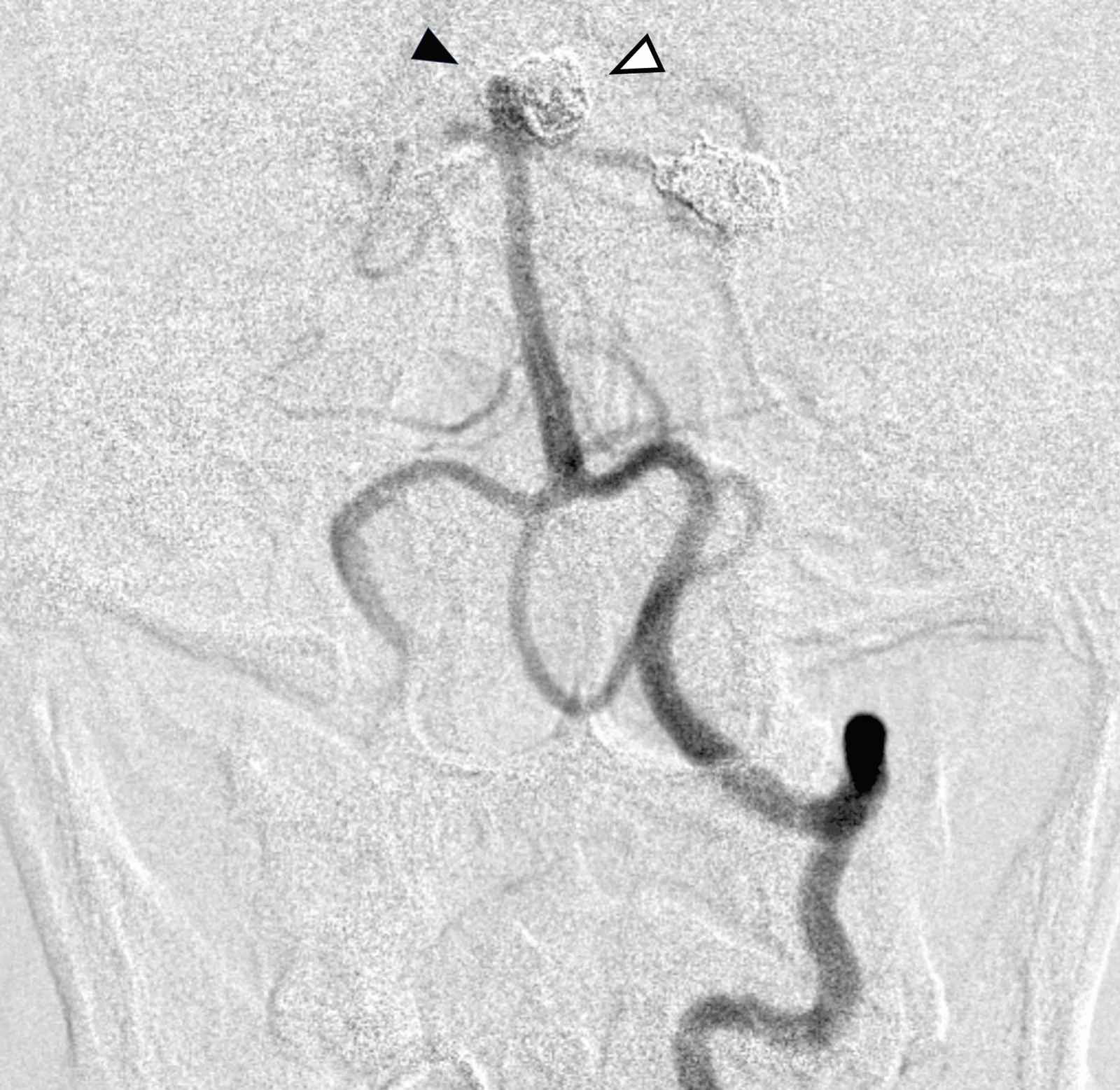
Patient 1’s previously stent-coiled basilar apex aneurysm involving the left posterior cerebral artery, showing recanalization (black arrowhead) and coil compaction (white arrowhead).

Patient 2 is a smoker who initially presented to us with a subarachnoid hemorrhage from a ruptured 19 × 21 mm basilar tip aneurysm. This aneurysm had been treated with coil occlusion 8 years earlier, and she had also been unreliable with follow-up. She was treated again with coil occlusion of the aneurysm and recovered from her hemorrhage. On post-discharge follow-up, she was demonstrated to have further coil compaction and dome recanalization at two-year follow-up (Figure 2). We initially planned for flow diversion into the dominant posterior cerebral artery from the basilar artery, but her proximal anatomy was too tortuous. Given her prior rupture and progressive growth, she was also evaluated for retreatment.

**Figure 2:**
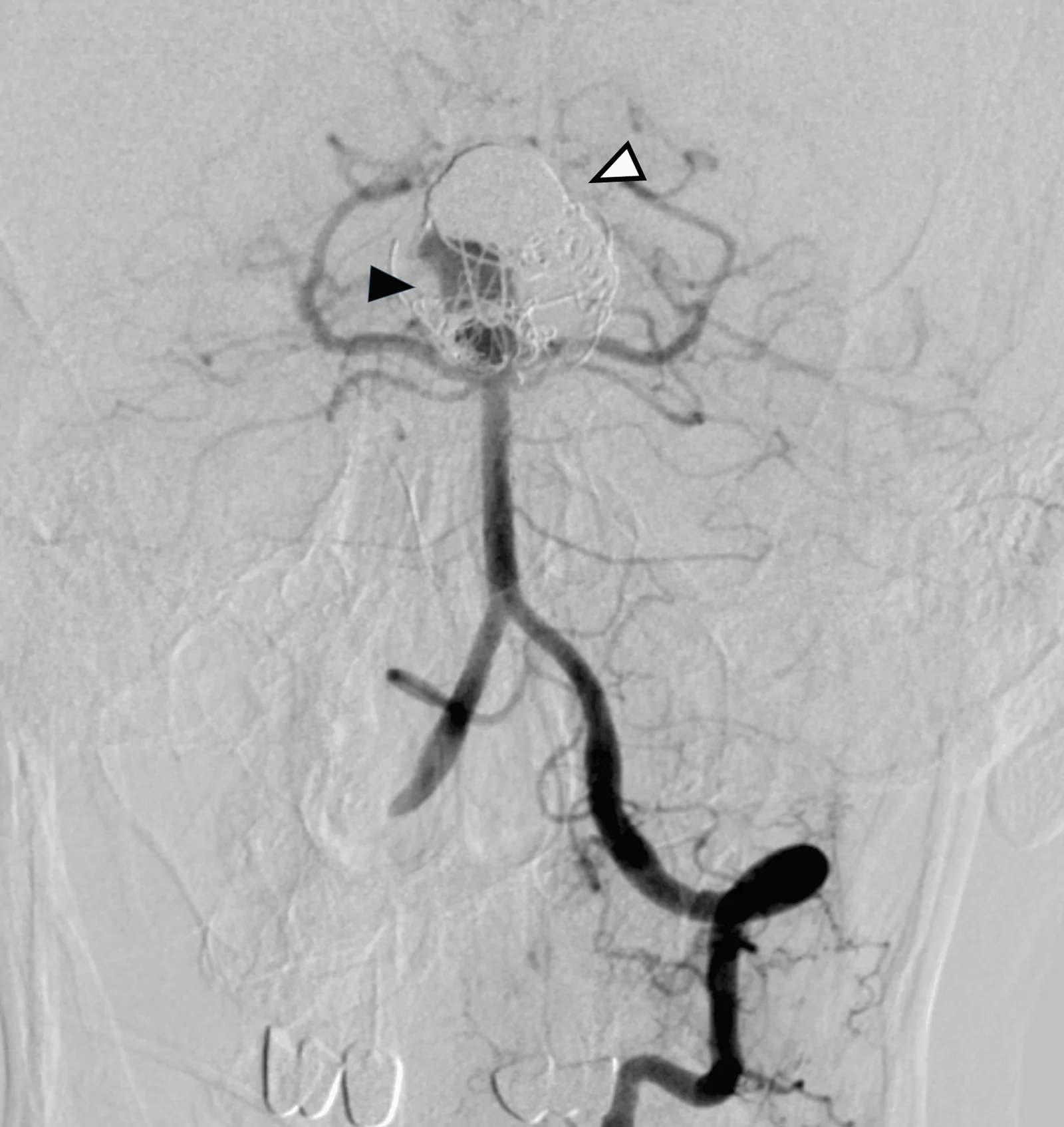
Patient 2’s previously coiled basilar apex aneurysm with recanalization of the dome (black arrowhead) and coil compaction (white arrowhead). This aneurysm was initially coiled 8 years earlier and received additional coils after the patient presented to our institution with rupture.

Crucially, preoperative angiography demonstrated the patency of at least one posterior communicating artery in each patient. Patient 1 had a left posterior communicating artery, and patient 2 had posterior communicating arteries present bilaterally.

Both patients received the same treatment: a subtemporal approach to basilar deconstruction. The subtemporal trajectory provides very limited access to CSF cisterns for brain relaxation. This is a particular concern in those who have had a prior aneurysm rupture, as access to the quadrigeminal cistern may yield little CSF. For this reason, a lumbar puncture is performed for diversion of 30 cc of CSF immediately prior to positioning in the operating room. A skin flap pedicled on the parietal branch of the superficial temporal artery and the posterior auricular artery is taken down along with the temporalis muscle. A small temporal craniotomy is made with a high-speed drill and extended to be flush with the middle fossa floor. The dura is opened, and dissection proceeds subtemporally. Dynamic, non-fixed retraction is used on the inferior temporal lobe to minimize brain manipulation during exposure. As the tentorial incisura is identified, the ambient cistern is opened and CSF is aspirated. The 4th cranial nerve and the P2 segment of the posterior cerebral artery are identified and followed to the basilar apex. To optimize visualization, the tentorium is cauterized and cut to facilitate caudal access into the posterior fossa and to the basilar trunk. The basilar artery is dissected, and the perforators are defined.

Of note, the perforators arising from the basilar artery are not evenly distributed along its course. There is a dense area of perforators in the mid-to-distal basilar artery. However, immediately before the apical quadrifurcation, there is a perforator-free zone (Figures 3A-3B). In this region, the perforators transition from basilar-originating, horizontally traversing vessels to cranially traversing vessels arising from the posterior cerebral and superior cerebellar arteries [9, 10]. This region can be identified from a subtemporal approach, where the lateral trajectory allows some visualization behind the basilar artery to ensure a window for safe passage of a clip. Prior to clip placement, it is crucial to identify the basilar trunk, both superior cerebellar arteries and posterior cerebral arteries, and the perforator-free zone. Both patients tolerated the procedure well and were discharged home after a few days of observation per our elective craniotomy protocol. Both patients were continued on their home aspirin 81 mg for other vascular indications. In patient 1, the aneurysm stabilized and demonstrated progressive regression to occlusion, which has remained durable out to 9 years (Figures 4-5). In patient 2, the aneurysm regressed and has remained stable out to three-year follow-up (Figure 6).

**Figure 3:**
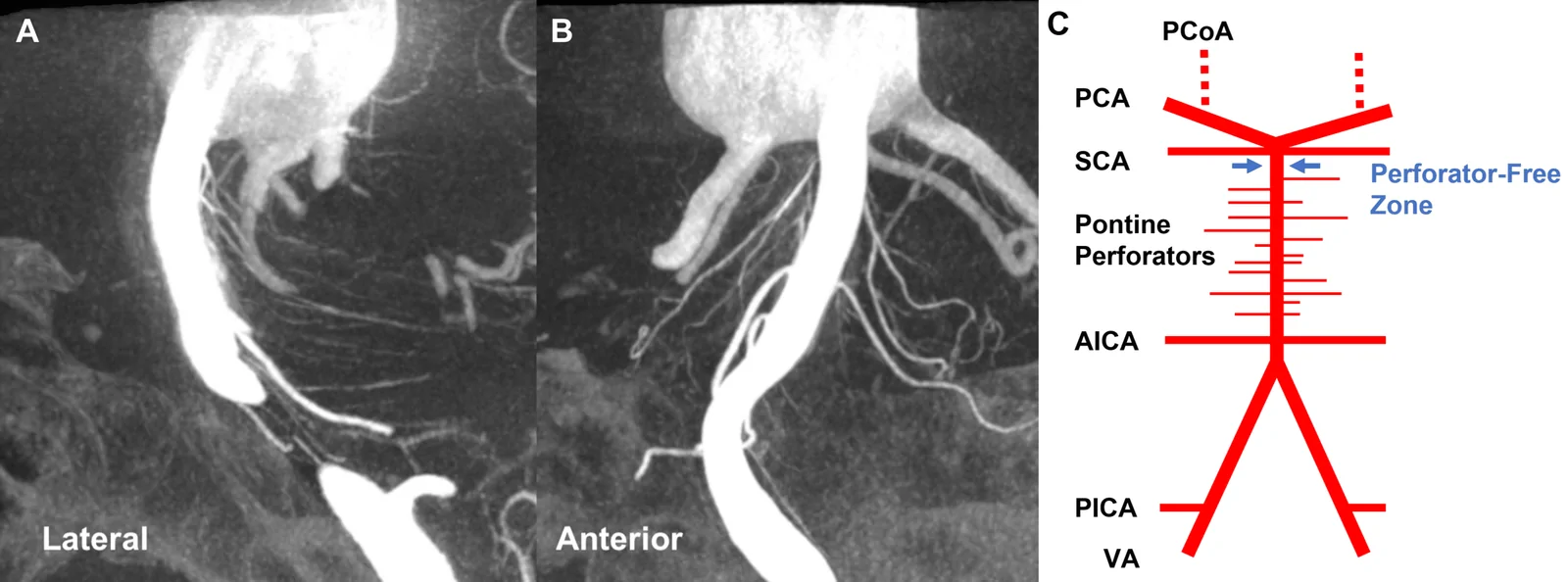
A representative lateral (A) and anteroposterior (B) maximum intensity praojection of the basilar perforators in an unrelated patient with a basilar apex aneurysm. Note the dense descending perforators to the pons along the basilar trunk, except for the few millimeters at the very top of the basilar artery before the takeoff of the superior cerebellar arteries. This is the perforator-free zone that allows safe passage of a microsurgical clip (blue arrows). The schematic (C) demonstrates a typical posterior circulation with the VA, PICA, AICA, pontine perforators, SCA, PCA, and PCoA. The perforator-free zone is shown as a disconnection point, allowing the lower part of the vertebrobasilar system to be supplied through the vertebral arteries and the upper part to be supplied through the posterior communicating arteries. AICA: anterior inferior cerebellar arteries; VA: Vertebral arteries; PICA: Posterior inferior cerebellar arteries; SCA: Superior cerebellar arteries; PCA: Posterior cerebral arteries; PCoA: Posterior communicating arteries.

**Figure 4:**
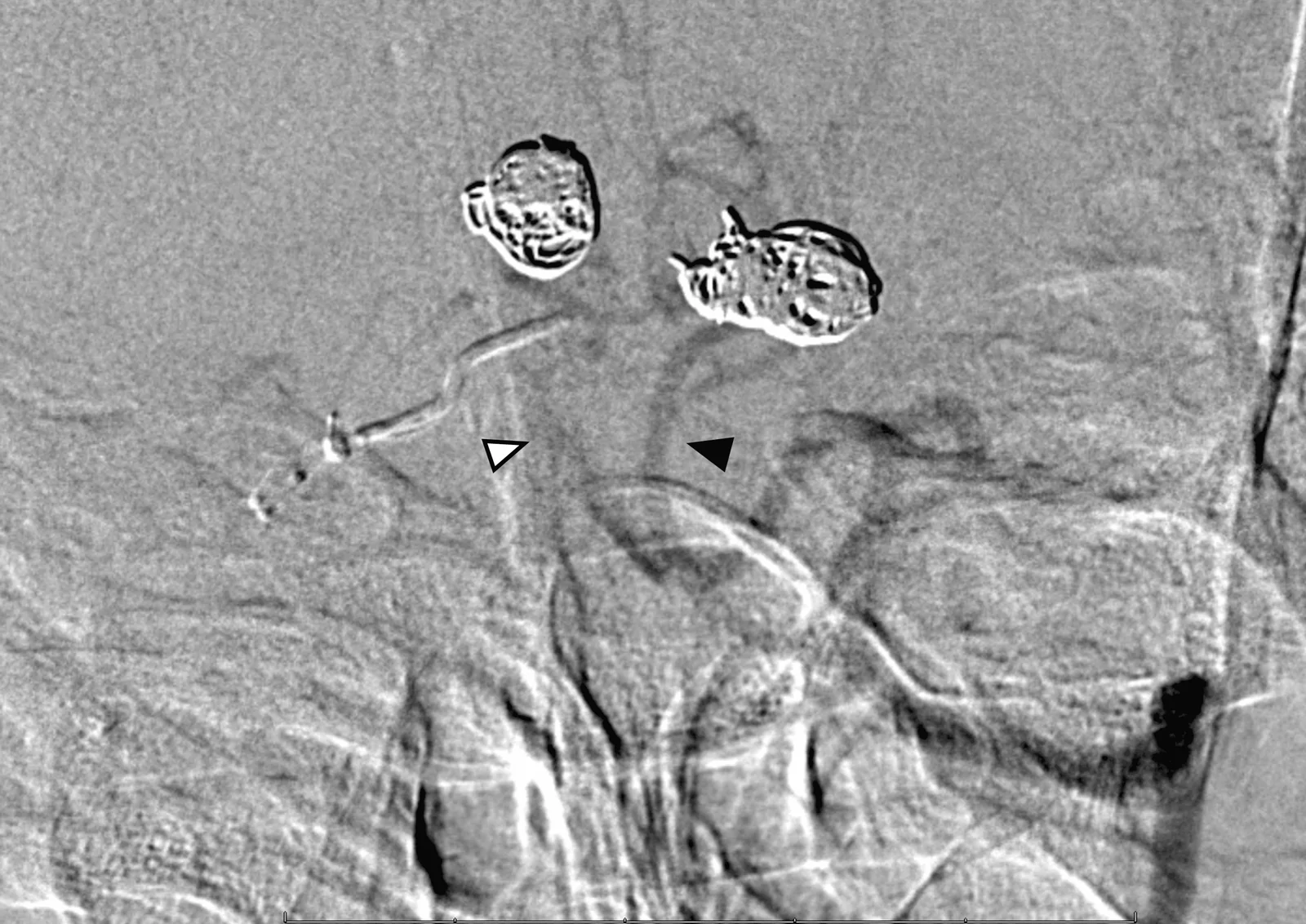
Patient 1 with an anteroposterior projection of a left vertebral artery injection demonstrating occlusion of the distal basilar trunk and no flow to the basilar apex aneurysm. There is expected minimal flow within the basilar trunk (white arrowhead), where only the perforators require filling, while the majority of the flow is directed to the left posterior inferior cerebellar artery (black arrowhead).

**Figure 5:**
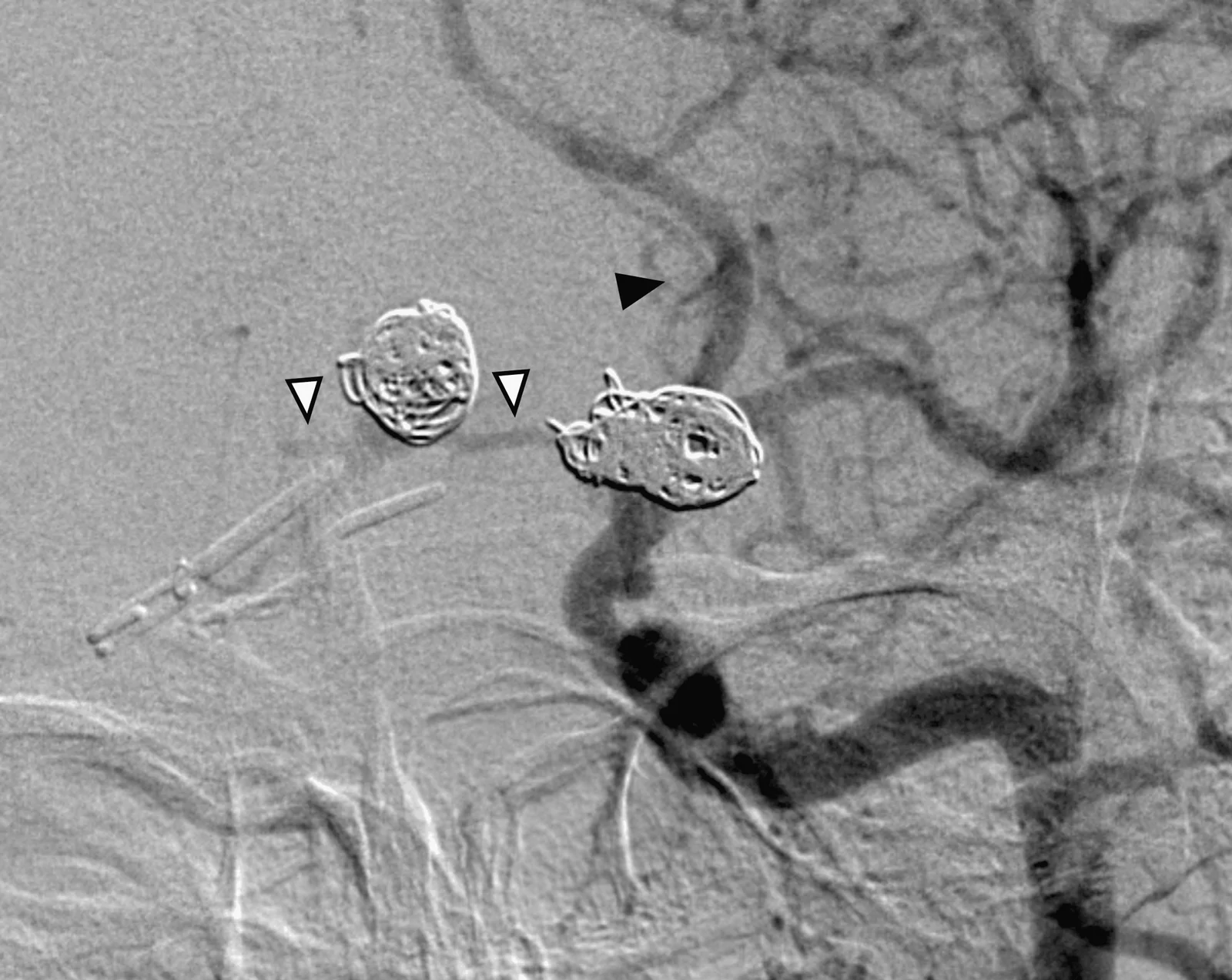
Patient 1 with a left common carotid artery injection demonstrating filling of the anterior circulation and cranial posterior circulation, including the left and right precommunicating segments of the posterior cerebral arteries (white arrowheads) and the left distal posterior cerebral artery (black arrowheads), through the left posterior communicating artery. The aneurysm still opacifies but with insufficient flow to sustain it. Note that there is no caudal filling of the basilar trunk despite filling of both precommunicating posterior cerebral arteries due to the microsurgical clip.

**Figure 6:**
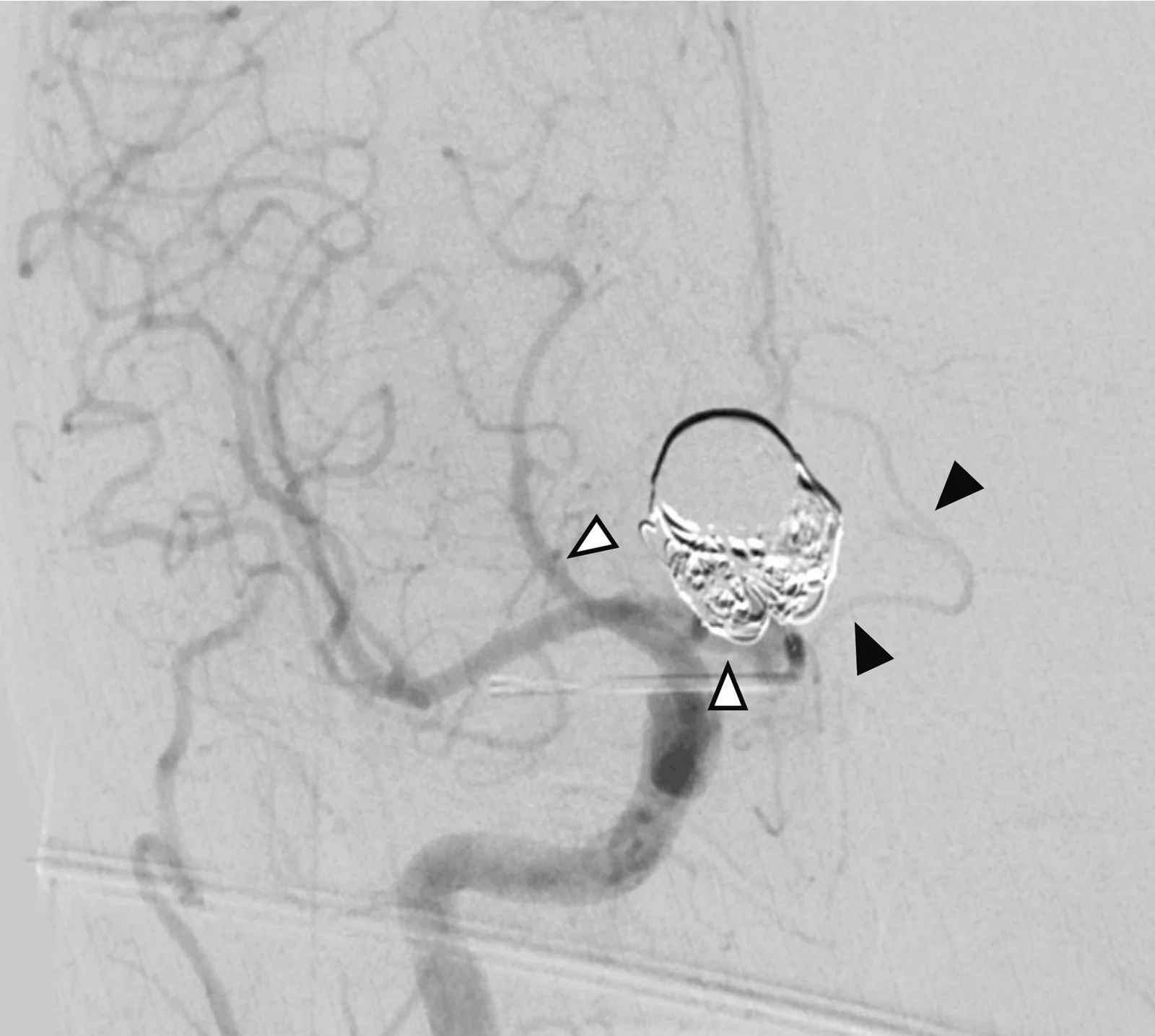
Patient 2’s follow-up right common carotid artery angiogram demonstrating flow through the right posterior communicating artery to the right posterior cerebral artery (white arrowheads) and the left posterior cerebral artery (black arrowheads). Note that there is no flow caudal to the microsurgical clip, demonstrating interruption of the basilar artery between its apex and trunk.

## Discussion

There are several strategies to manage recurrent basilar apex aneurysms that grow despite treatment, albeit each with its own limitations [4, 5, 7]. In endovascular occlusion-based strategies using coils and endosaccular flow disruptors, adding more material and maintaining ongoing surveillance can mitigate rupture risk [5]. However, by definition, refractory aneurysms typically do not achieve durable results with this strategy. Being lost to follow-up also presents a significant risk with this palliation-oriented strategy, as seen initially in both of our cases. With flow diversion-based strategies that reduce flow to the dome and presumably reduce rupture risk, diverting the dominant flow to the aneurysm can interrupt necessary flow to other branches at the basilar apex, cover perforators, and necessitate potent antiplatelet medications. Results are also limited by the complex “endoleak” created by multiple sources of flow outside the flow diverter within the basilar apex or aneurysm [11]. Direct surgical clipping of the aneurysm can be made difficult by the endoluminal bulk of prior interventions, as well as the tendency for these aneurysms to sit high in the posterior fossa and impact themselves into the base of the brain [7]. Reconstructive surgical strategies, such as cerebrovascular bypass, dramatically increase the complexity of the intervention and add another potential site of failure. In fact, it has been reported that the increased flow from a bypass can perpetuate an aneurysm and promote its rupture [12]. Conversely, surgical deconstruction by Hunterian ligation of the distal basilar artery is a well-established, often overlooked, and effective way to manage these aneurysms.

Among the myriad endovascular options to treat this disease, disconnection is another possibility that we report here. However, intraluminal vascular occlusion devices generally occupy a much larger segment of the artery than an externally applied, 1-mm-wide aneurysm clip. In the setting of dense basilar perforators immediately proximal to the perforator-free segment, this strategy may be unwise. Another option is staged occlusion of both vertebral arteries. In this case, however, the posterior communicating artery or arteries must then provide flow to the entire posterior circulation, as opposed to just the posterior cerebral artery territories [13].

A physiologic perspective bears mentioning. The dominant inflow to the basilar apex is from the basilar trunk and often projects blood directly toward the dome of the aneurysm. Most of the blood flow passing into the basilar apex ultimately flows into the posterior cerebral arteries. These arteries serve not only a larger volume of tissue than the superior cerebellar arteries but also provide flow largely to the cortex, which has a disproportionately high demand for blood flow compared with that of white matter and the cerebellum [14]. This interruption immediately proximal to the basilar apex redirects blood flow from the aneurysm dome in two beneficial directions: either blood is shunted away from the dome entirely (in the case of a posterior communicating artery supplying the ipsilateral posterior cerebral artery) or into an almost tangential path across the neck and not into the dome (in the case of a posterior communicating artery supplying the contralateral posterior cerebral artery across the basilar apex). The physics of blood flow patterns that promote aneurysm formation and growth are complex and not well understood, but this change appears to reduce flow stress on the aneurysm dome and promote a beneficial physiologic change.

## Conclusions

Recurrent aneurysms of the posterior circulation that are refractory to initial treatment present a major and ongoing challenge to those who treat these patients. While there is no shortage of treatment options given the complicated anatomy of the basilar apex, it remains unclear which option is best suited for each patient. Here, we present a technical note on surgical disconnection of the distal basilar artery as a treatment option, a simple yet effective strategy that can lead to long-term pacification of these difficult-to-treat aneurysms. We believe that deconstruction eliminates the direct arterial jet into the aneurysm, allowing flow changes that lead to permanent cessation of growth, decreased bleeding risk, and reduced mass effect.
